# *Arabidopsis* AUGMIN8 Contains Two Independent Microtubule Association Domains

**DOI:** 10.1002/cm.70068

**Published:** 2025-11-10

**Authors:** Naveen K. Chana, Timothy Cioffi, Sidney L. Shaw

**Affiliations:** ^1^ Department of Biology Indiana University Bloomington Indiana USA

**Keywords:** AUGMIN, microtubule nucleation, plant microtubules

## Abstract

Plant cells create a plasma membrane‐associated network of microtubules that are nucleated by γ‐tubulin ring complexes primarily through microtubule‐dependent microtubule nucleation (MDMN). This dynamic array organizes into specific patterns in response to developmental and environmental cues to influence primary cell wall construction. The molecular mechanisms directing the creation of cortical microtubule array patterns are largely unknown. The hetero‐octameric AUGMIN complex facilitates mitotic spindle formation by associating γ‐tubulin ring complexes with existing spindle microtubules and creating parallel branched microtubules through MDMN. AUGMIN8, the key linker protein connecting the AUGMIN complex to the parent microtubule, is encoded by a paralogous family of QWRF genes in flowering plants. Members of the QWRF family are distinguished by an unstructured N‐terminal half encoded in a single 5′ exon. We hypothesize that the QWRF paralogs form interchangeable AUGMIN microtubule binding subunits that confer specific roles to the AUGMIN complex in mitotic and non‐mitotic microtubule arrays. We identify four QWRF family members expressed in *Arabidopsis* hypocotyl cells and investigate the sites of QWRF interaction with cortical microtubules using transient transformation of fluorescently tagged constructs in the heterologous *Nicotiana benthamiana* system. We show that full‐length QWRF8 and QWRF4 associate with non‐mitotic, cortical microtubules as distributed puncta where QWRF8 shows evidence for two independent sites of microtubule association. Sequence comparisons and in vivo assay with homologous fragments from QWRF1, 2, 4, and 5 define a shared N‐terminal conserved microtubule association domain. We additionally identify protein regions leading to the formation of microtubule‐associated “QWRF bodies” potentially linked to discontinuous localization on microtubules. We identify the “QWRF” protein motif as a conserved domain associating the AUGMIN8 paralogs with AUGMIN6, part of the larger AUGMIN complex.

## 1 Introduction

Cortical microtubules in acentrosomal plant cells form specific array patterns at the cell cortex in response to developmental and environmental cues (Elliott and Shaw 2018). The array patterns direct cellulose deposition into the nascent wall encoding specific material properties required for morphogenesis (Baskin 2001; Cyr and Palevitz 1995; Green 1962). Microtubules are nucleated from γ‐tubulin ring complexes (γTuRC) typically associated with existing cortical microtubules (Chan et al. 2009; Murata and Hasebe 2007; Nakamura et al. 2010; Shaw et al. 2003). This microtubule‐dependent microtubule nucleation (MDMN) has been observed in both parallel and antiparallel orientations to the parent microtubule and can be either branched or unbranched (Chan et al. 2009; Shaw and Lucas 2011; Yagi et al. 2018). Cortical array microtubules subsequently release from the γTuRC but remain laterally associated with the cell cortex (Nakamura et al. 2010; Shaw et al. 2003). The polymers exhibit a hybrid form of treadmilling, leading to cortical translocation and interactions with other microtubules (Shaw et al. 2003; Ehrhardt and Shaw 2006). How specific sites are selected for cortical microtubule nucleation and how nucleation contributes to cortical array patterning in response to developmental and environmental cues remain critical open questions in biology.

The AUGMIN protein complex plays an essential role in eukaryotic mitotic and meiotic spindle formation (Goshima et al. 2008; Lawo et al. 2009; Petry et al. 2011, 2013; Uehara et al. 2009). The octameric complex anchors the γTuRC to spindle microtubules and promotes the formation of branched, parallel nucleation events (Hsia et al. 2014; Kamasaki et al. 2013; Song et al. 2018; Verma and Maresca 2019). The AUGMIN complex also appears in some acentriolar and non‐mitotic animal arrays (Cunha‐Ferreira et al. 2018, 2016; Sánchez‐Huertas et al. 2016; Schweizer et al. 2021; Viais et al. 2021; Zhang et al. 2024). All eight AUGMIN subunits were identified in flowering plants and the AUGMIN complex is essential for acentrosomal plant mitotic spindle formation (Ho et al. 2011; Hotta et al. 2012). The AUGMIN complex also appears in non‐mitotic plant cells and was shown to influence the microtubule nucleation angle (Liu et al. 2014). These data suggest that AUGMIN could serve as a central factor in directing microtubule nucleation for plant acentriolar microtubule arrays.

The hetero‐octameric AUGMIN protein complex in animal cells associates with the parent microtubule through the AUGMIN8 (HAUS8/HICE1) subunit with possible assistance from AUGMIN6 (Wu et al. 2008; Zhu et al. 2008). Protein interaction and structural studies show the C‐terminal half of AUGMIN8 associating with the larger AUGMIN complex, interacting directly with AUGMIN6 and AUGMIN7. The N‐terminal half of AUGMIN8 appears to be highly unstructured (Gabel et al. 2022; Hsia et al. 2014; Song et al. 2018; Travis et al. 2023; Zupa et al. 2022). Association with microtubules was reported for the N‐terminal half of AUGMIN8 (1–140 AA) (Wu et al. 2008) and at least one phosphorylation site in the unstructured sequence confers a regulatory property for microtubule binding (Tsai et al. 2011).

Genetic experiments in the model flowering plant, *Arabidopsis thaliana* , indicated that ENDOSPERM DEFECTIVE 1 (EDE1) was critical for spindle formation (Pignocchi et al. 2009). Noting a conserved amino acid motif, EDE1 was associated with a family of nine related “QWRF” genes (Albrecht et al. 2010; Pignocchi et al. 2009). EDE1 (QWRF5) shows limited homology with animal AUGMIN8 proteins and, through biochemical and molecular genetic experiments, was shown to be a mitotic paralog of AUGMIN8 (Lee et al. 2017). Promoter swapping experiments with other QWRF genes indicated the specificity of EDE1 (QWRF5) for mitotic spindles (Lee et al. 2017). Work with other QWRF family members revealed that QWRF1 mutants show defects in cotyledon chloroplast development (Albrecht et al. 2010) and double mutants for QWRF1 and 2 produce morphological phenotypes described in floral tissues (Ma et al. 2021). Together with the work in Liu et al. (2014) showing AUGMIN localization at the plant cell cortex, these studies provide evidence that flowering plants may have evolved different AUGMIN8 paralogs to allow for AUGMIN function under different cell cycle or developmental contexts.

The *Arabidopsis* hypocotyl serves as a leading model for microtubule studies in flowering plants and in connecting cortical microtubule array defects to plant growth phenotypes (Baskin et al. 1994; Collett et al. 2000; Gendreau et al. 1997; Mathur 2004). We, therefore, have focused this work on identifying QWRF genes with possible hypocotyl function for analysis. The association of the mitotic AUGMIN complex with microtubules in animal cells requires multiple regulatory steps including AUGMIN8 phosphorylation and loss of ALPHA‐IMPORTIN binding (Kraus et al. 2023; Tsai et al. 2011; Ustinova et al. 2023). We hypothesize that AUGMIN complex association with microtubules in non‐mitotic plant cells is also highly regulated and subject to a wider range of regulatory inputs governing where, when, or in what configuration (e.g., branched vs. unbranched) microtubules are nucleated. With this study, we begin to address how proposed non‐mitotic AUGMIN8 paralogs encoded by the QWRF gene family associate with plant cortical microtubules.

## 2 Results

### 2.1 Modular Architecture of *Arabidopsis* QWRF Protein Family

**FIGURE 1:**
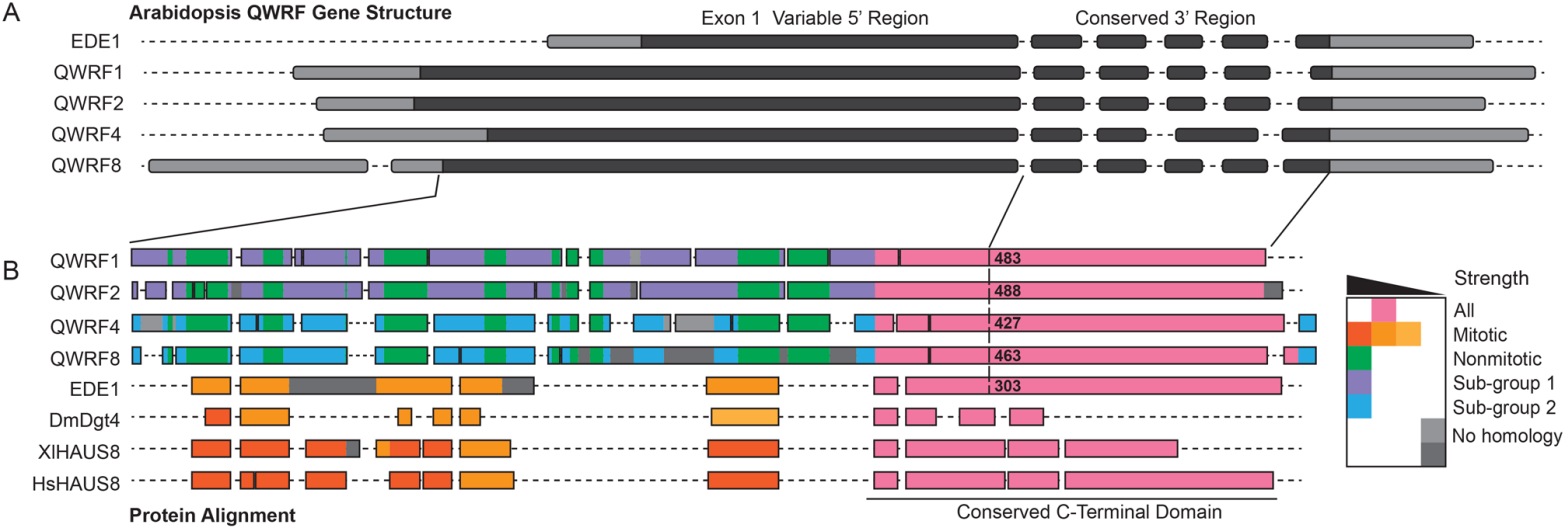
*Arabidopsis* AUGMIN8 paralogs have a modular structure. (A) Gene structures for members of the *A. thaliana* QWRF protein family encoding the mitotic QWRF5 (EDE1) and four paralogs expressed in non‐mitotic hypocotyl cells QWRF8 (AUG8), QWRF4, QWRF1, and QWRF2. Light gray = 5′/3′ UTR, dark gray = protein coding exons. (B) Homologous protein sequence domains for QWRF family proteins and AUGMIN8 orthologs from *D. melanogaster* (Dgt4), *X. laevis* (HAUS8), and humans (HAUS8). Exons are mapped to the orthologous protein alignment from A to B with colors representing relative strength of protein homology between groups and the amino acid position for the junction encoded by the 5′ exon labeled for each protein.

We identified four *Arabidopsis* QWRF gene family members (QWRF1, 2, 4, and 8) with similar hypocotyl expression levels in publicly available datasets (Chapman et al. 2012). These putative non‐mitotic AUGMIN8 paralogs share a conserved overall gene structure with the mitotic EDE1 (QWRF5) (Figure 1A). Family members show strong sequence conservation across the 3′ exon set, predicted to encode the C‐terminal domain that associates AUGMIN8 with the larger AUGMIN complex (Gabel et al. 2022; Travis et al. 2023; Zupa et al. 2022). Each QWRF gene contains a single, 5′ exon predicted to encode unstructured protein domains that associate the AUGMIN complex with the parent microtubule. The 5′ exon retains a nearly identical portion of the conserved C‐terminal domain in all cases (Figure 1A,B). Alignment of QWRF protein sequences using ClustalX (Larkin et al. 2007) indicated QWRF1‐2 and QWRF4‐8 as sub‐paralogous pairs, sharing conserved regions but independent of the mitotic EDE1 (QWRF5) protein (Figure 1B). All plant QWRF N‐terminal protein domains share conserved serine‐ and threonine‐rich sequences and arginine‐ and lysine‐rich sequences not found in EDE1 (QWRF5) or animal HAUS8/HICE1 orthologs (Figure 1B). These data suggest a modular gene architecture maintaining a conserved C‐terminal domain for AUGMIN complex integration and variable N‐terminal domains, encoded by a monolithic 5′ exon predicted to associate the AUGMIN complex with existing microtubules.

### 2.2 Expression of *Arabidopsis* QWRF Proteins and Microtubule Markers in *Nicotiana benthamiana*

**FIGURE 2:**
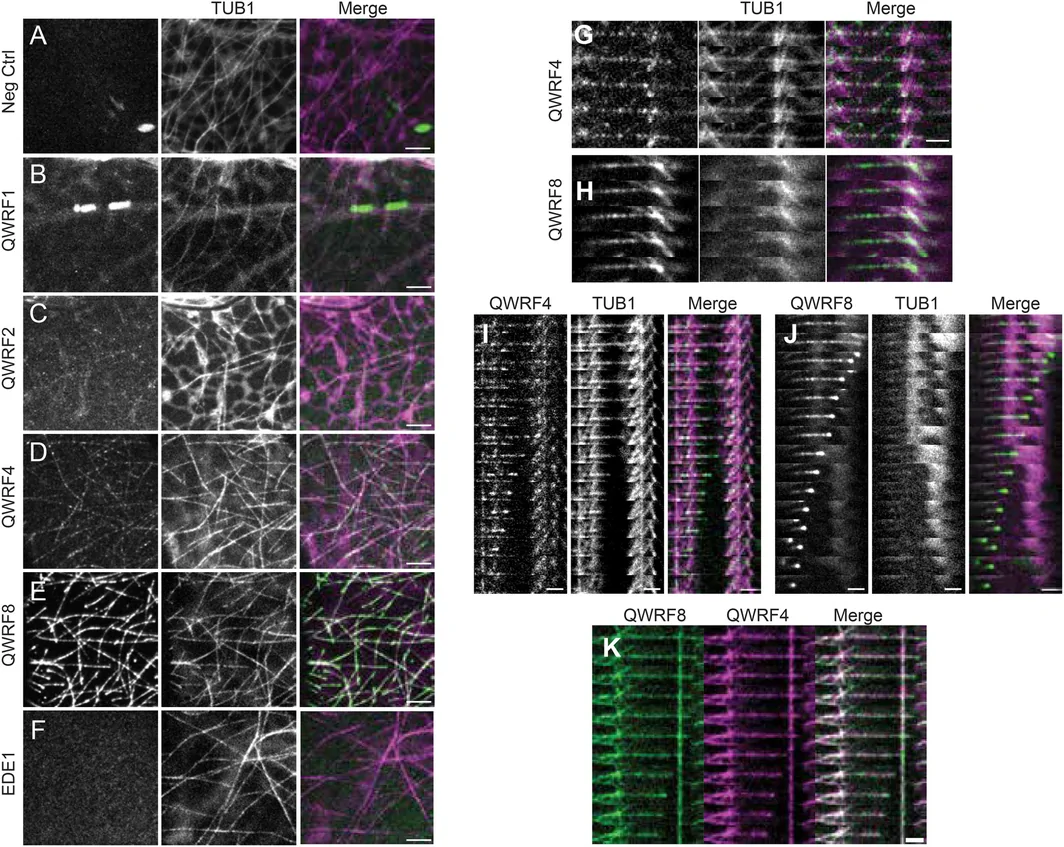
Co‐expression of full length QWRF genes and a microtubule marker in a heterologous plant system. Spinning disk confocal microscopy of *N. benthamiana* leaf pavement cells after inoculation with *A. tumifacians* carrying binary plasmids for (A) no vector control, (B) mNEON‐QWRF1, (C) QWRF2‐mScarlet, (D) QWRF4^cDNA^‐mNEON6xHis, (E) QWRF8^cDNA^‐mNEON6xHis, and (F) EDE1^cDNA^‐mNEON6xHis. Leaf tissue was co‐inoculated with *A. tumifacians* for co‐expression of 35s:mScarlet‐TUB1 or 2x35s:mNEON‐TUB1 (A–F). Scale bar = 5 μm. Time‐lapsed montage of (G) QWRF4 and (H) QWRF8 associated with microtubule lattice over 12 s at 3 s intervals showing shifting of individual foci. Time‐lapsed montage showing the retention or accumulation of (I) QWRF4 or (J) QWRF8 at the end of a depolymerizing microtubule plus‐end over 42 s of microtubule depolymerization shown at 3 s intervals. All QWRF constructs are pseudocolored green and microtubules pseudocolored magenta. (K) Co‐expression of QWRF8‐mNeonGreen and QWRF4‐mScarlet shown as merged time‐lapsed images at 3 s intervals over 3 min showing areas of independent and overlapping co‐localization. Scale bar in G–K = 2 μm.

We hypothesized that the N‐terminal regions of QWRF proteins confer distinct regulatory or microtubule binding characteristics to the AUGMIN complex. The unstructured N‐terminal half of AUGMIN8 has not been structurally resolved to date and the isolated protein has not appeared in in vitro microtubule association studies. Therefore, to investigate our hypothesis, we used *Agrobacterium* to transiently co‐express full‐length *QWRF* and *BETA‐TUBULIN1 (TUB1)* genes linked to fluorescent proteins in *N. benthamiana* leaf epidermal cells (Figure 2). Genomic and cDNA clones for *QWRF1, 2, 4, 8*, and *EDE1 (QWRF5)* were created using the same CaMV‐35s constitutive promoter with either an mNEON‐Green (Shaner et al. 2013), mNEON‐Green6xHis, or mScarlet1 (Bindels et al. 2017) fluorescent protein sequence fused to the QWRF 3′ end. Genomic TUB1 clones, using the same 35s promoter, were fused to an N‐terminal fluorescent protein of opposite color. Spinning disk confocal microscopy of live control (no *QWRF* gene) cells showed plastid autofluorescence and cortical microtubules with unincorporated fluorescent fusion protein highlighting cellular membrane and cytosol (Figure 2A). Fluorescence levels for both QWRF and tubulin transgenes varied independently from cell to cell in all cases producing a range of protein concentrations and imaging conditions.

QWRF1 and QWRF2 remained diffuse in the cytoplasm and surrounding the endoplasmic reticulum, with only occasional puncta co‐localizing to microtubules observed at very high expression levels (Figure 2B,C). In contrast, QWRF4 and QWRF8 (AUG8) localized to cortical microtubules over a wide range of expression levels, forming discontinuous or punctal patterns with little cytoplasmic signal at high expression levels (Figure 2D,E). EDE1 (QWRF5) was diffuse in the cytoplasm and showed no co‐localization with cortical microtubules (Figure 2F).

To investigate the nature of the punctal association, we performed time‐lapse imaging of QWRF4 and QWRF8 puncta. Image series indicated lateral travel or “wobble” over spatial scales of less than 1 μm over a 12 s imaging window (Figure 2G,H). QWRF4 and QWRF8 were further observed to accumulate on depolymerizing microtubule ends (Figure 2I,J). We interpret the movement and intensity to suggest that both QWRF4 and QWRF8 form structures larger than a single molecule or associate in linked proximity on the microtubule lattice. We observed no evidence for microtubule nucleation events at puncta and no obvious change in the density of microtubules in the cortical array. These observations collectively suggest that QWRF4 and QWRF8 associate with microtubules as multimeric units but do not reconstitute functional AUGMIN complexes related to microtubule nucleation.

Noting the similar dynamics and discontinuous distributions for QWRF4 and QWRF8 on microtubules, we wanted to determine if they formed co‐localizing complexes on the microtubules or acted independently of each other. Co‐expression of both probes, using differently colored fluorescent proteins, revealed that QWRF4 and QWRF8 show regions of overlapping label and distinct independent regions of presumed microtubule association. These data suggest that QWRF4 and QWRF8 do not obligately or uniformly co‐associate on the microtubule lattice and can occupy different regions of the same microtubule (Figure 2K).

### 2.3 Domain Analysis of *Arabidopsis* QWRF8 Microtubule Association

**FIGURE 3:**
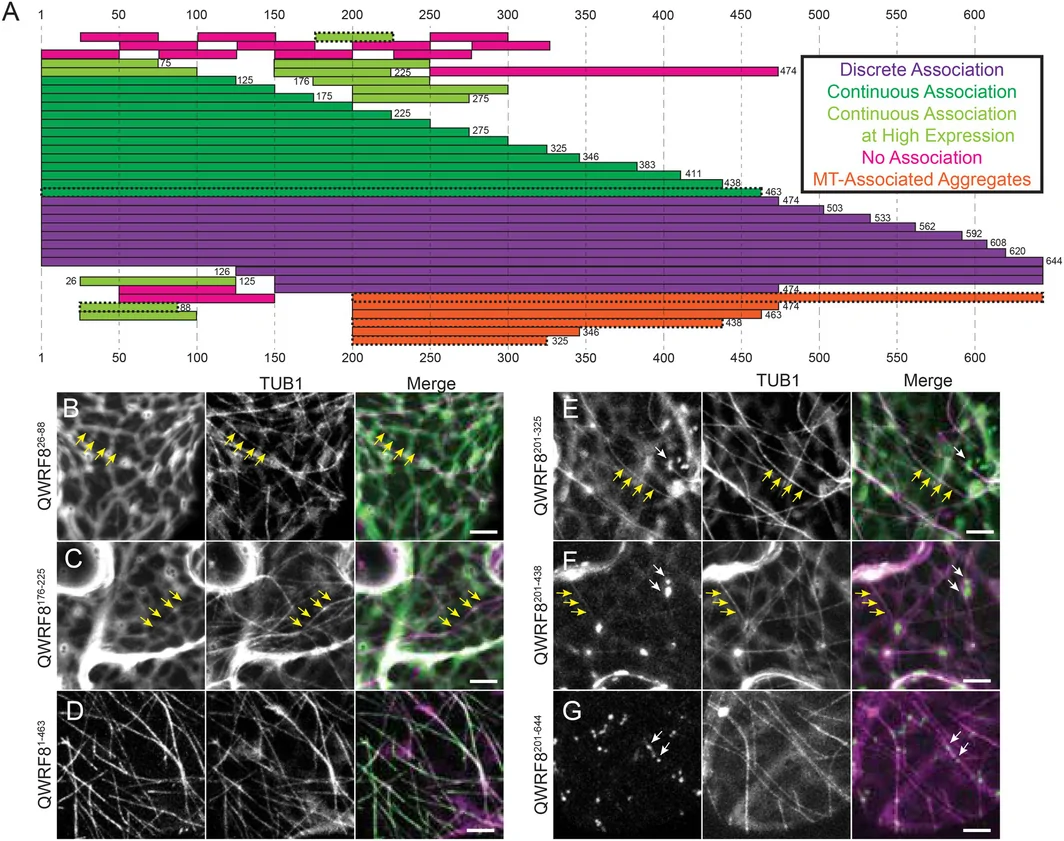
Determination of QWRF8 microtubule association domains. (A) Representation of QWRF8 protein fragments tested for microtubule association in *N. benthamiana* transient co‐expression experiments highlighted by color for result. (B–G) Representative images of QWRF8‐mNEON fragments, indicated by dashed outlines in (A), and mScarlet‐TUB1. (B) Image showing just detectable co‐localization (yellow arrows) for the first minimal microtubule association domain QWRF8^26–88^ and (C) a minimal second association domain QWRF8^176–226^ encoded in the first monolithic 5′ exon. (D) N‐terminal fragment encompassing exon 1 QWRF8^1–463^ containing both microtubule association sites exhibits predominantly continuous microtubule association. (E) Continuous microtubule labeling (yellow arrows) and formation of small microtubule associated QWRF bodies (white arrow) observed for fragments QWRF8^201–325^ lacking the first microtubule association domain. Longer fragments initiating at AA 200 create larger microtubule associated QWRF bodies (F) and a shift away from continuous microtubule localization (G).

The full‐length QWRF8 protein consistently labeled non‐mitotic cortical microtubules in the transient expression assays exhibiting a discontinuous localization (Figure 2E). To determine the protein sequence required for microtubule association, we transiently expressed truncated cDNA clones using a 35s constitutive promoter and an mNEON‐Green or mNEON‐Green6xHis fluorescent protein sequence fused at the 3′ end (Figure 3). *N. benthamiana* leaves were co‐inoculated with mScarlet1‐TUB1 and imaged using spinning disk confocal microscopy. Initial screens of the N‐terminal region using either consecutive 50 AA fragments, overlapping every 25 AA, or fragments of increasing 25 AA length (Figure 3A), revealed two independent regions conferring microtubule association. Fragments starting beyond AA 225 did not localize to microtubules, independent of expression level. Using data from the initial screening, we identified QWRF8^26–88^ as a minimal association domain showing detectable colocalization at high expression levels (Figure 3B). Fragments using AA 50–125 and AA 50–150 showed no evidence for microtubule localization.

Expression of QWRF8 fragments beginning at AA 200 showed microtubule colocalization with a minimum second association site found for QWRF8^176–225^ (Figure 3C). We observed evidence for microtubule association in the absence of AA 176–200, but only when additional C‐terminal sequence was included (Figure 3A). Therefore, we predict a minimum second associating domain to appear within QWRF8^201–225^ but do not observe association without surrounding sequence. These observations establish two independent in vivo microtubule association domains encoded by the first 5′ exon of QWRF8.

### 2.4 *Arabidopsis* QWRF8 Domains Show Microtubule‐Associated Aggregation

In contrast to the discontinuous labeling of microtubules observed for full‐length QWRF8 (Figure 2E), the minimal association domains appeared predominantly as a continuous distribution on cortical microtubules (Figure 3B,C). To determine what part of the QWRF8 sequence controls this localization property, we expressed QWRF8 fragments of increasing 25 AA length initiating at the N‐terminus (Figure 3A). We observed a distinct transition from continuous to discontinuous microtubule labeling at AA 474 of the 644 AA QWRF8 protein (Figure 3A). Extending from QWRF8^1–463^ to QWRF8^1–474^ was sufficient to convert from continuous to discrete localization. This observation suggests that the unstructured QWRF8 N‐terminus, encoded by the first exon (AA 1–463), shows continuous binding in the absence of the conserved C‐terminal domain. We expressed the first monolithic QWRF8^1–463^ exon (Figure 3D) and verified continuous association. These observations indicate that the region between AA 463 and 474, forming the juncture with the conserved C‐terminus, constitutes a structural or regulatory region sufficient to switch the microtubule association from continuous to discontinuous.

**FIGURE 4:**
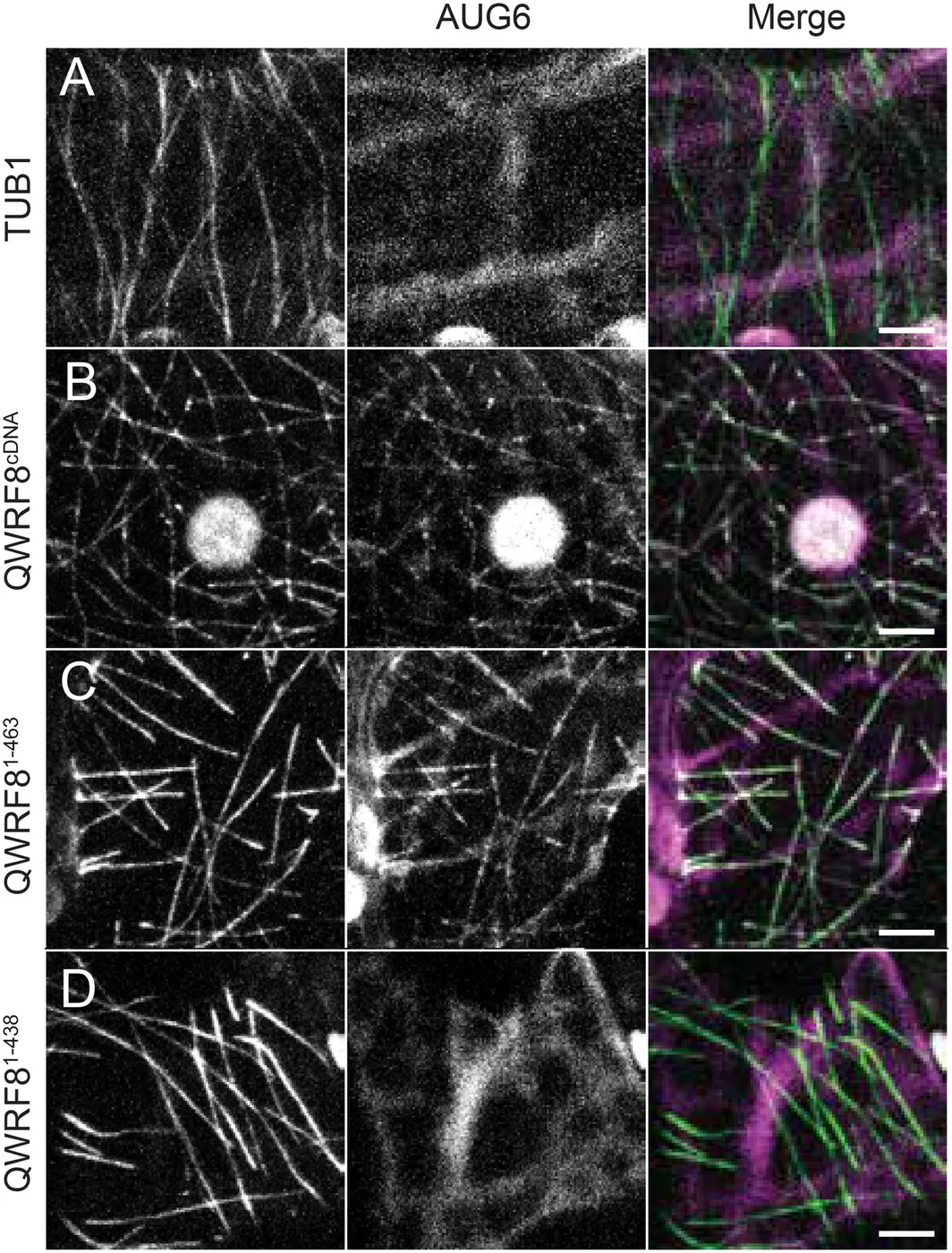
Association of AUGMIN6 to cortical microtubules by QWRF8. (A) Expression of full‐length AUG6‐mScarlet with mNEON‐TUB1 in *N. benthamiana* leaf epidermal cells indicated no AUGMIN6 localization to cortical microtubules. (B) Co‐expression of full‐length QWRF8^cDNA^‐mNEON with AUG6‐mScarlet produced overlapping discontinuous association of both proteins to cortical microtubules. (C) Co‐expression of the QWRF8^1–463^ first with AUG6 resulted in continuous co‐localization of both proteins to cortical microtubules. (D) Expression of progressively shorter QWRF8 fragments indicates that the region between AA438 and AA463 is required to associate AUGMIN6 to cortical microtubules. Scale bar = 5 μm.

We tested the requirement for the first microtubule association site to create the discontinuous microtubule labeling by expressing fragments starting at AA 125 or AA 150 (beyond the first association site) and reaching to AA 474 or AA 644 (Figure 4A). These fragments showed discontinuous labeling in line with full‐length QWRF8 (Figure 2E) indicating that the first association site is not required for the discontinuous labeling. The fragments used to identify the minimal second microtubule association site showed continuous localization at high expression levels for fragments starting at AA 200 and extending to AA 275 (Figure 3A). Expressing fragments starting at AA 200 and extending beyond AA 300 produced an unusual phenomenon. These QWRF fragments exhibited microtubule association but additionally produced larger cytoplasmic aggregates or “QWRF‐bodies” (Figure 3E–G). The size of the fluorescent QWRF‐body generally increased progressively with increasing length of the QWRF8 fragments. In time‐lapse observations, these structures showed discontinuous association with microtubules or moved in the cytoplasmic streams with stops on the microtubules (Movie S1). Fragments near the boundary at AA 474, established for the continuous to discontinuous association (i.e., QWRF8^201–438^, QWRF8^201–463^, and QWRF8^201–474^), showed increasingly larger fluorescent QWRF‐bodies. Extension to the full C‐terminus (QWRF8^201–664^) created large aggregates with fewer obvious microtubule interactions (Figure 3G). These observations indicate that QWRF8 has a capacity for either interacting with itself or forming an aggregate with other cytoplasmic components. The aggregation is minimized or regulated by the second microtubule associating domain and the sequence just beyond the first exon.

### 2.5 Minimum AUGMIN8 Region for Association With AUGMIN6

We postulated from the gene structure of the QWRF family that interaction with the larger octameric AUGMIN complex likely occurs after the first monolithic exon, QWRF8^1–463^ (Figure 1). To examine our hypothesis, we created a full‐length genomic construct of the *Arabidopsis* AUGMIN6 protein, predicted to interact with the C‐terminal portion of AUGMIN8 as part of the larger AUGMIN protein complex (Gabel et al. 2022; Travis et al. 2023; Zupa et al. 2022). Using the transient *N. benthamiana* system, we first co‐expressed 35s:AUG6‐mScarlet with 35s:mNEON‐TUB1 and observed only cytoplasmic AUG6 localization (Figure 4A). When co‐expressed with QWRF8^cDNA^‐mNEON, AUG6‐mScarlet associated with microtubules, co‐localizing exactly with QWRF8 puncta (Figure 4B). Similar to prior observations (Lee et al. 2017), these data indicate that we cannot observe AUG6 localizing to microtubules by itself or through association with the native *N. benthamiana* AUGMIN complex, but the *Arabidopsis* QWRF8 brings *Arabidopsis* AUG6 to the cortical microtubules. To test our hypothesis that AUG6 association occurs through the conserved C‐terminal region of the AUG8 protein, we co‐expressed AUG6‐mScarlet with the first exon of QWRF8^1–463^‐mNEON (Figure 4C). We observed, counter to our hypothesis, that AUG6 associated with QWRF8^1–463^ continuously on the microtubules. To determine the minimum site of AUG6 interaction, we identified a 25 AA span (QWRF8^438–463^) near the 3′ end of exon 1 with strong homology across all QWRF family members. Co‐expression of AUG6 with QWRF8^1–438^ showed no AUG6 association (Figure 4D), indicating that a conserved region in the first exon anchored by the “NRxxQWRF” motif is sufficient for interactions with AUG6 on the cortical microtubules. We further observed that the association of AUG6 with QWRF8 at the cell cortex does not require QWRF8 to appear in a discontinuous localization pattern.

### 2.6 Paralogous Sequence Conservation of QWRF Functional Domains

**FIGURE 5:**
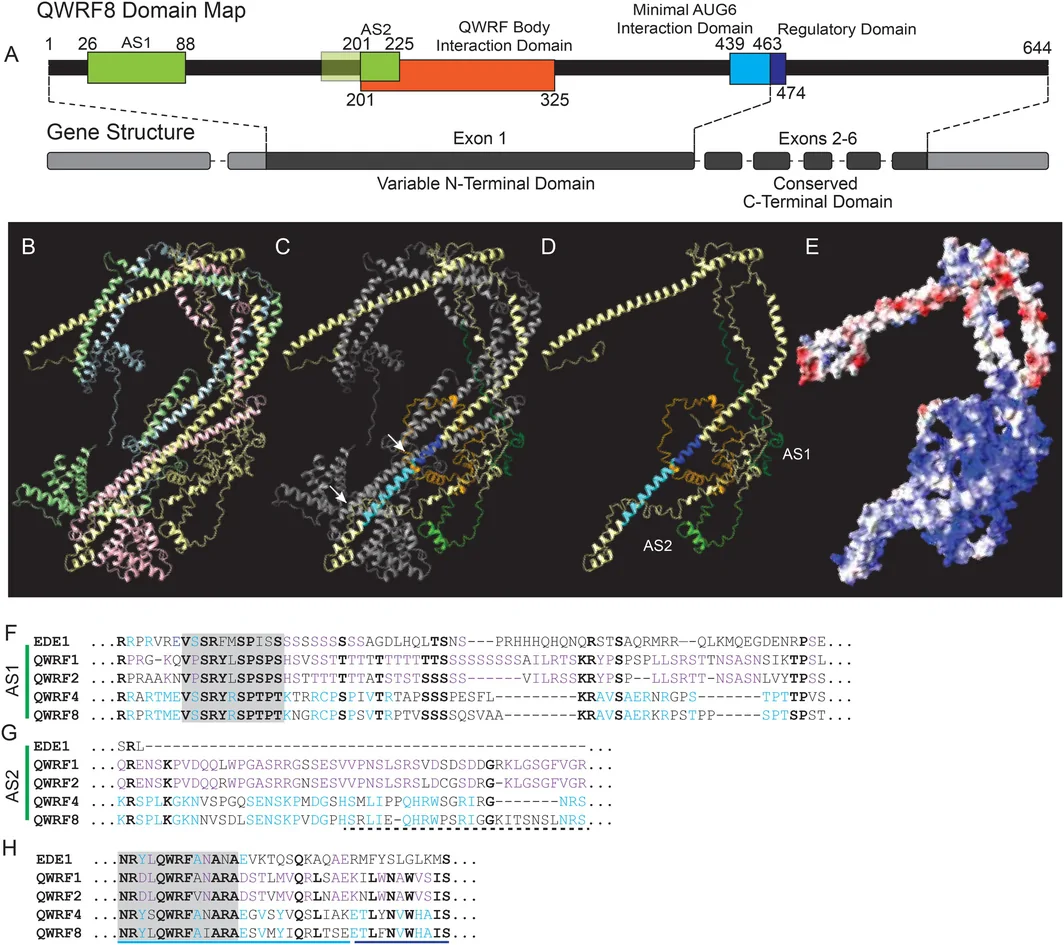
Mapped QWRF8 domains for microtubule and AUGMIN6 association. (A) Linear representation of the QWRF8 644 AA protein sequence with minimal microtubule association domains (green), the region forming QWRF bodies (orange), the minimum domain for AUGMIN6 association (cyan), and the transition region from continuous to discontinuous QWRF microtubule labeling (blue). Functional protein domains are mapped to the QWRF8 gene structure where light gray = 5′/3′ UTR and dark gray = protein coding sequence. (B–D) Protein structure models threading *A. thaliana* proteins through human AUGMIN complex based on data from Gabel et al. (2022). (B) Predicted structure of the AUGMIN sub‐tetramer composed of AUG2 (blue), AUG6 (pink), AUG7 (green), and AUG8 (yellow) showing the QWRF8 C‐terminus interacting with the other subunits with the intrinsically disordered N‐terminus outside of the sub‐tetramer structure. (C) The sub‐tetramer structure with QWRF8 in yellow and mapped functional domains color coded as in (A). (D) QWRF8 isolated from the sub‐tetramer with color coding as in (C). (E) QWRF8 calculated surface charge indicating the microtubule association domains reside within a larger domain of exposed positive charges (blue) separated from the C‐terminal helices with predicted negative (red) and neutral (white) charges. (F–H) Protein sequence alignments for mapped domains in (A) for EDE1, QWRF8, QWRF4, QWRF1, and QWRF2 with color coding for homology across paralogous pairs. Residues conserved across at least four of the proteins are bolded. Gray shading in (F) and (H) indicate minimal conserved motifs.

Our evaluation of QWRF8 identified multiple functional domains including two microtubule association sites (AS1 and AS2), a sequence leading to the formation of QWRF bodies, a region controlling discontinuous localization, and a minimal sequence for associating AUG6 to QWRF8 on cortical microtubules (Figure 5). These functional domains provide evidence that the monolithic 5′ exon, different for each QWRF gene, encodes the sequence elements that associate the protein with microtubules and carries other possible functional information (Figure 5A). To place these domains into a structural context, we threaded the *Arabidopsis* QWRF8 protein into the animal HAUS8 protein structure, in complex with the AUGMIN microtubule associating tetramer (from Gabel et al. 2022). The unstructured N‐terminus contains a small portion of AS2 forming an alpha‐helix (Figure 5B–D). Highlighting the QWRF8 protein within the tetramer, and identifying the functional domains within QWRF8 (Figure 5C,D), suggests that the microtubule association domains are exposed and would associate the tetramer with the QWRF8 C‐terminal extension facing away from the microtubule. The minimal sequence required for AUG6 association appears as part of an alpha‐helix immediately adjacent to, or in contact with, AUG6 predicted from the threaded model (Figure 5C). The small domain shown to regulate the switch from continuous to discontinuous association, sitting at the junction encoded by the 5′ exon, forms a continuation of the alpha helix predicted for AUG6 association, but provides no immediate information about how it affects microtubule binding. The protein region producing QWRF bodies is unstructured and predicted to be somewhat internal to the domains associating with the microtubule (Figure 5D). The unstructured region of QWRF8, where the microtubule association domains reside, is predicted to display a strong aggregate positive charge (Figure 5E).

To determine if the functional domains identified in QWRF8 appear in the other QWRF paralogs from this study, we aligned protein sequences to identify possible homologies (Figure 5F–H). We found that QWRF8 microtubule AS1 (Figure 5F) shows strong conservation across both the non‐mitotic and mitotic AUGMIN8 paralogs toward the N‐terminal part of the domain. The protein sequences diverge after this motif into sub‐paralogous groupings where EDE1, QWRF1 and 2 introduce a string of 9–25 S/T residues. Little homology appears in the remainder of the domain suggesting “VxSRYxSPTPT” as a common motif and minimal microtubule association domain for the QWRF gene family. In contrast, homology with QWRF8 microtubule AS2 (Figure 5G) was weak for QWRF4 and not recognizable for QWRF1, QWRF2, or EDE1. This observation suggests that the second microtubule association domain may be unique to QWRF8 or that the non‐mitotic AUGMIN8 paralogs have different sequences related to this region for microtubule association.

Sequence comparisons indicate that the association of QWRF8 to AUG6 occurs through a highly conserved domain (Figure 5H) that includes the namesake “QWRF” motif. Interestingly, the predicted alpha helical domain distal to the QWRF motif, shown to alter the character of the localization, shows more limited homology between non‐mitotic paralogs and we find no recognizable homology with EDE1 sequence, despite the high conservation of the QWRF domain (Figure 5H). The region indicated to produce QWRF‐bodies exhibited only weak homology between sub‐paralogous pairs and virtually no recognizable homology with EDE1, similar to the microtubule AS2 domain.

### 2.7 Functional Conservation of Domains in *Arabidopsis* QWRF Proteins

**FIGURE 6:**
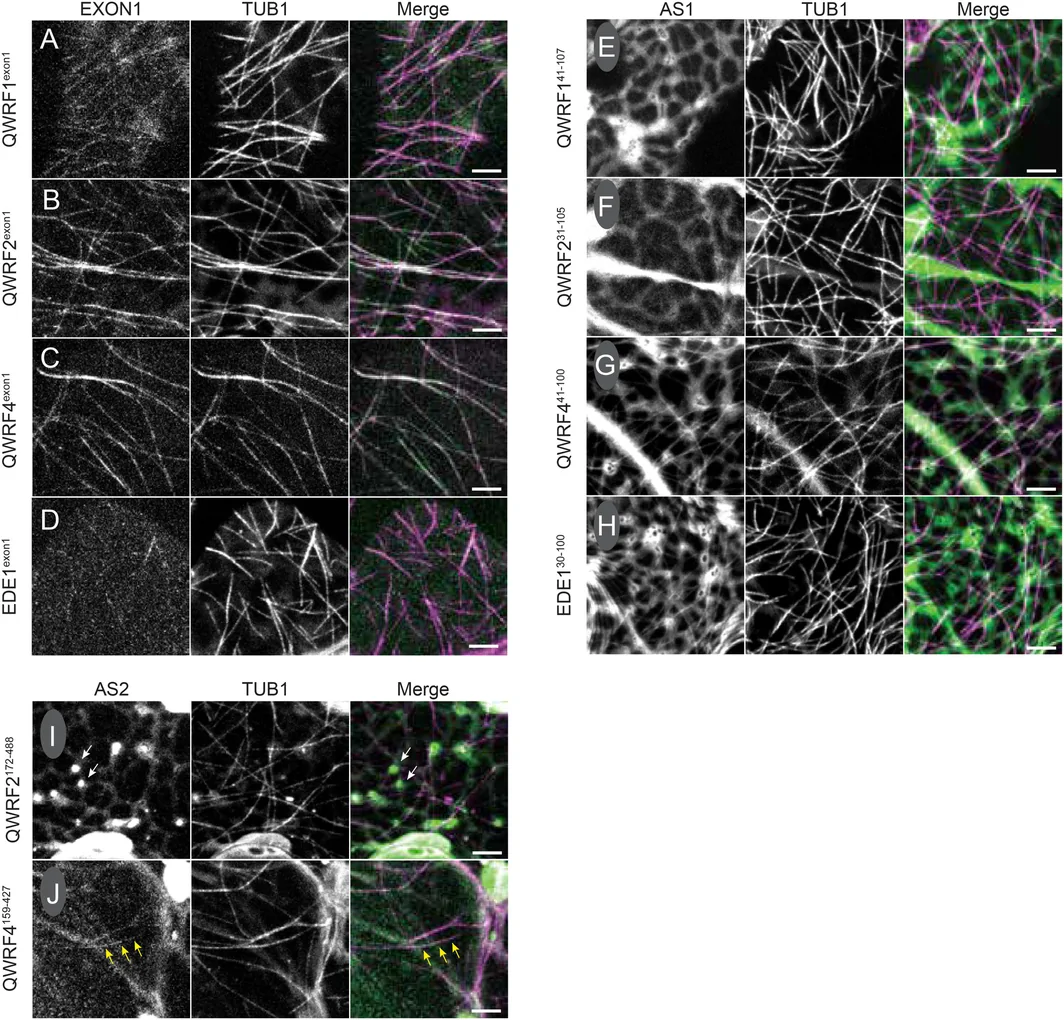
Microtubule association of N‐terminal domains from QWRF paralogs. Expression of (A) QWRF1^exon1^‐mNEON, (B) QWRF2^exon1^‐mNEON, (C) QWRF4^exon1^‐mNEON6xHis, and (D) EDE1^exon1^‐mNEON6xHis with 35s:mScarlet‐TUB1 in *N. benthamiana*. QWRF1^exon1^ and EDE1^exon1^ display weak microtubule co‐localization. QWRF2^exon1^ and QWRF4^exon1^ display continuous microtubule co‐localization. (E–H) Sequence regions most homologous to the QWRF8 microtubule association domain I were fluorescently tagged with mNEON and transiently co‐expressed with 35s:mScarlet‐TUB1 in *N. benthamiana*. No consistent microtubule association was observed for (E) QWRF1^41–107^ or (F) QWRF2^31–105^, where (G) QWRF4^41–100^, and (H) QWRF5/EDE1^30–100^ produced weak continuous co‐localization with microtubules. Expression of fragments hypothesized to contain a second N‐terminal microtubule association domain for (I) QWRF2^172–488^ produced only large microtubule‐associated aggregates (white arrow) and weak, but consistent co‐localization for (J) QWRF4^159–427^ (yellow arrows). Scale bars = 2 μm.

To determine if the predicted domains for other QWRF proteins result in microtubule association, we cloned and expressed the first exon of QWRF1, 2, 4, and EDE1 as a fluorescent fusion in *N. benthamiana* leaf epidermal cells co‐expressed with a fluorescent tubulin marker (Figure 6). In the absence of the conserved C‐terminal domain, we observed weak continuous cortical microtubule labeling for QWRF1, 2, and 4 (Figure 6A–C). Co‐localization of the EDE1 construct with microtubules was only observed under extremely high expression levels and was only visible against bright (assumed bundled) microtubules (Figure 6D).

We identified AS1 QWRF8^26–88^ as a first minimal microtubule association domain with sequence conservation among QWRF1, 2, 4, 5, and 8 family members. To determine if this domain is conserved for microtubule association, we cloned the corresponding QWRF1^41–107^, QWRF2^31–105^, QWRF4^41–100^, and EDE1/QWRF5^30–100^ fragments for transient expression assays. Consistent with results for the full‐length protein, and opposite to results using only the first exon, we did not observe consistent co‐localization for QWRF1 or QWRF2 fragments even at very high expression levels (Figure 6E,F). Expression of QWRF4^41–100^ (Figure 6G) produced weak, continuous microtubule association, similar to QWRF8^26–88^. Expression of EDE1/QWRF5^30–100^ produced variable but identifiable microtubule co‐localization similar to EDE1/QWRF5^exon1^ (Figure 6H). Together, these experiments confirm microtubule association via the first exon and provide evidence for microtubule AS1 functioning for the plant mitotic EDE1 and QWRF4.

Sequence comparisons did not indicate obvious regions of homology for the second microtubule association site found in QWRF8 (Figure 5G). Moreover, we found that the much shorter N‐terminal half of EDE1 appears to be missing this region altogether. To ask if the other QWRF family members contain a second microtubule association domain, we cloned fragments for QWRF2 and 4 starting at the end of the QWRF8 microtubule AS1 and extending to the end of the first exon (QWRF2^172–488^ and QWRF4^159–427^). The QWRF2^172–488^ fragment (Figure 6I), which is nearly identical to QWRF1 (Figure 5G), formed large aggregates that either moved in cytoplasmic streams or discontinuously colocalized with microtubules, much like the QWRF‐bodies observed with QWRF8 fragments starting at AA201 and extending past AA300. QWRF4^159–427^ (Figure 6J) was primarily cytoplasmic with limited but consistent continuous microtubule colocalization observed at high expression levels on (presumably) microtubule bundles.

### 2.8 QWRF Association to Spindle Microtubules in Induced Mitotic Cells

**FIGURE 7:**
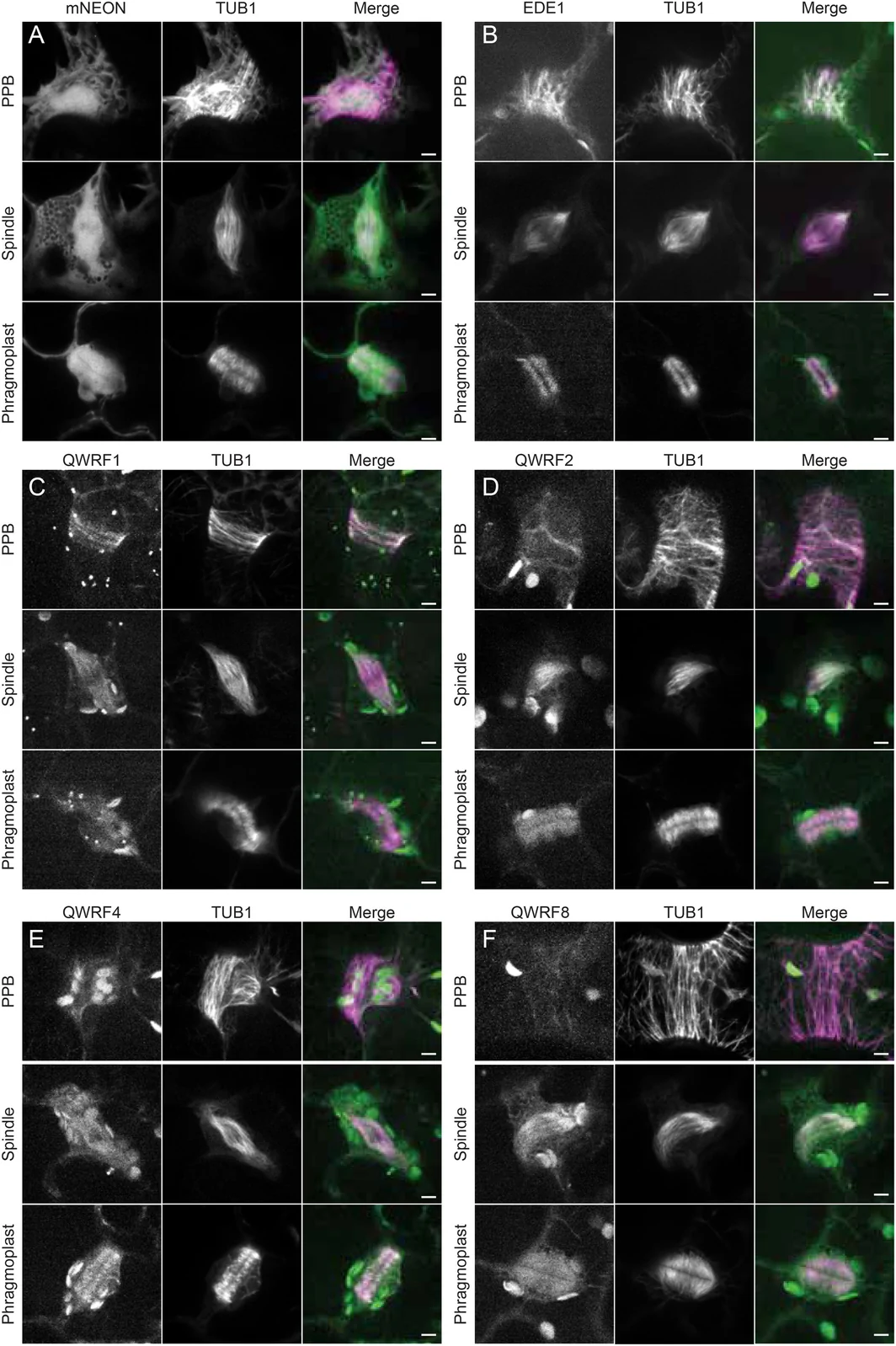
Localization of QWRF proteins in mitotic microtubule arrays. Constructs expressing (A) free mNEON control, (B) EDE1^cDNA^mNEON6xHis, (C) mNEON‐QWRF1, (D) QWRF2‐mScarlet, (E) QWRF4^cDNA^‐mNEON6xHis, and (F) AUG8^cDNA^‐mNEON6xHis were transiently expressed under the CaMV 35s promoter with 35s:AtCYCD3;1 and 35s:mScarlet‐TUB1 or 2x35s:mNEON‐TUB1 in *N. benthamiana* leaf epidermal pavement cells. Image projections for each construct include the microtubules forming a pre‐prophase band (PPB), the mitotic spindle, and the phragmoplast array. (A) Control free mNEON expression highlights regions of cytoplasmic thickness and protein exclusion with no specific microtubule association. (B) EDE1 association with PPB, spindle, and phragmoplast microtubules. (C) QWRF1 showed limited interaction with PPB and spindle microtubules. (D) QWRF2 showed weak colocalization with microtubules at the spindle midzone. (E) QWRF4 displayed weak phragmoplast association, but no spindle or PPB association (F) QWRF8 displayed no association with PPB or phragmoplast microtubules with inconsistent association to spindle microtubules at high expression levels. Scale bar = 5 μm.

Prior work showed that transiently expressed EDE1 (QWRF5) associated with microtubules in the mitotic spindle (Lee et al. 2017). To determine if the putative non‐mitotic AUGMIN8 paralogs associate with plant mitotic microtubule arrays, we used methods from Xu et al. (2020) to induce mitosis in *N. benthamiana* leaf epidermal cells via co‐inoculation with *Agrobacterium* containing *Arabidopsis* cyclin D3 (CYCD3;1). Plant cells entering mitosis form a pre‐prophase band (PPB) of cortical microtubules as the interphase cortical array disassembles. The PPB disassembles during spindle formation and the phragmoplast array forms after anaphase to create the cellular partition for cytokinesis. Determining in vivo association of proteins with mitotic microtubules is made difficult by the reorganization of the plant cytoplasmic surrounding the arrays. Cells expressing free mNEON fluorescent protein with mScarlet1‐TUB1 showed the intensity distribution of the cytoplasm with reference to the PPB, spindle, and phragmoplast arrays (Figure 7A). Cells co‐inoculated for EDE1^cDNA^‐mNEON6xHis and mScarlet‐TUB1 expression revealed EDE1 co‐localizing with PPB microtubules (Figure 7B) and, as previously reported (Lee et al. 2017; Pignocchi et al. 2009; Xu et al. 2020), with spindle and phragmoplast microtubules.

We next assayed QWRF1, 2, 4, and 8 for association to microtubules in mitotic cells (Figure 7C–F). QWRF1‐mNEON (Figure 7C) showed just detectable association with PPB microtubules and limited evidence for spindle association with no clear association to phragmoplast arrays. The QWRF2‐mScarlet protein (Figure 7D) showed just detectable co‐localization with mitotic spindle arrays and no consistent association with PPB or phragmoplast. The QWRF4^cDNA^‐mNEON6xHis (Figure 7E) protein overlapped weakly with phragmoplast microtubules but showed no clear association with spindle or PPB microtubules. The QWRF8^cDNA^‐mNEON6xHis (Figure 7F) did not show consistent co‐localization to the mitotic arrays beyond concentration to spindle pole regions when very highly expressed (Figure 7A). Together, these data provide evidence that these plant AUGMIN8 paralogs do not consistently associate with mitotic microtubules but show limited interactions with arrays either early (i.e., QWRF1) or late (i.e., QWRF4) as cells are transitioning into and out of mitosis. Extending prior work (Pignocchi et al. 2009; Xu et al. 2020), these data indicate cell cycle dependent regulation of QWRF family microtubule association, differentiating mitotic from non‐mitotic family members.

**FIGURE 8:**
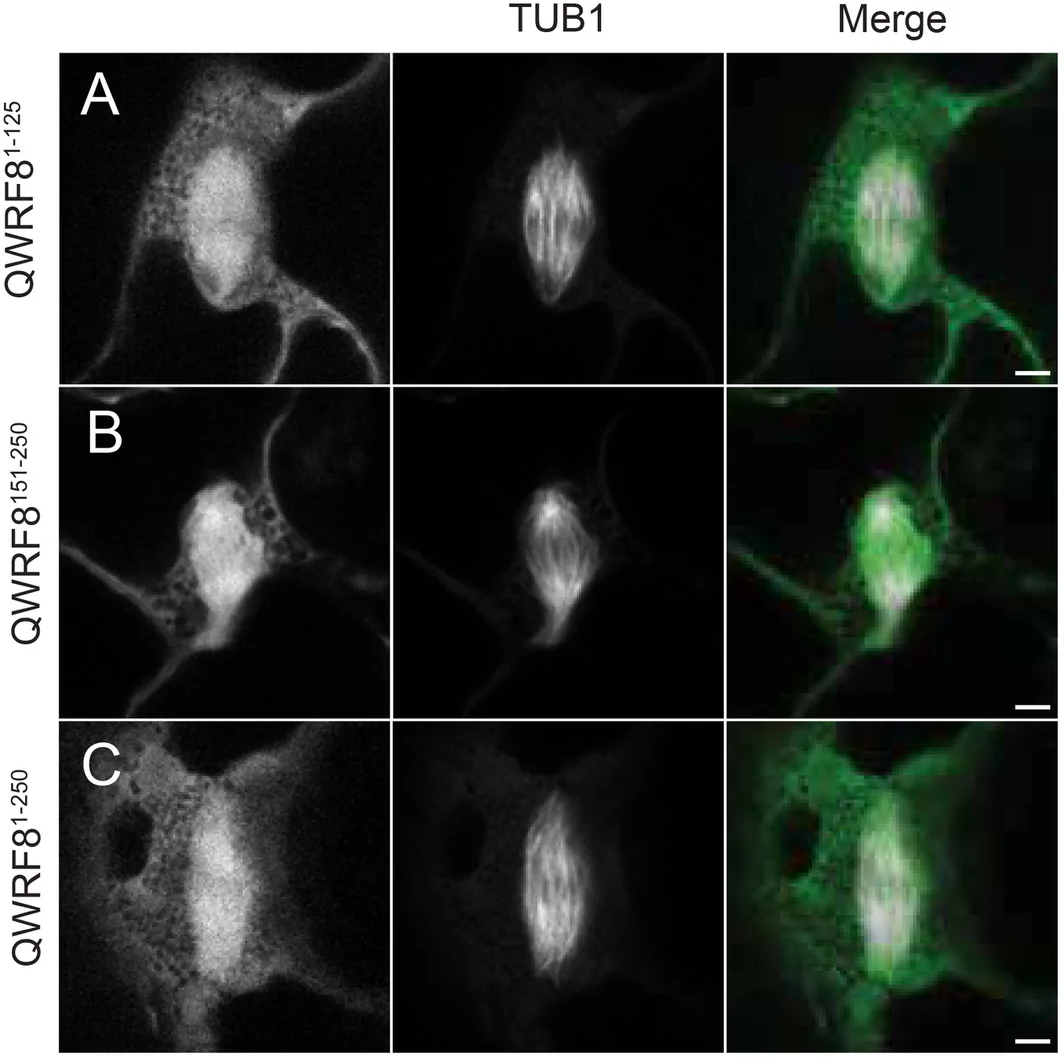
QWRF8 fragments do not specifically associate with mitotic spindle microtubules. Co‐expression of N‐terminal QWRF8 fragments including (A) only the first microtubule association domain QWRF8^1–125^mNEON, (B) only the second microtubule association domain QWRF8^151–250^‐mNEON, or (C) both microtubule association domains AUG8^1–250^mNEON with AtCYCD3;1 and mScarlet‐TUB1 in *N. benthamiana* indicated no specific association to mitotic spindle microtubules. Scale bar = 2.5 μm.

The full length QWRF8 protein showed inconsistent localization with mitotic spindle microtubules suggesting cell cycle regulation of QWRF8 association (Figure 7F). To determine if the regulation could be dissociated from either of the discovered QWRF8 microtubule association domains, we again used co‐inoculation with CYCD3;1 expressing constructs in *N. benthamiana* to induce mitotic arrays (Figure 8). Expression of QWRF8^1–125^ (association domain 1), QWRF8^151–250^ (association domain 2), or QWRF8^1–250^ (association domains 1 and 2) revealed no consistent association with spindle microtubules despite showing interactions with cortical array microtubules.

## 3 Discussion

### 3.1 A Modular Subunit for Plant AUGMIN Microtubule Binding

The AUGMIN complex plays a critical role in spindle formation in both plant and animal cells (Goshima et al. 2008; Ho et al. 2011; Hotta et al. 2012; Lawo et al. 2009; Uehara et al. 2009). AUGMIN localization within the spindle, and the structural properties leading to parallel branched nucleation, are critical for correctly orienting new spindle microtubules (Sánchez‐Huertas and Lüders 2015). The AUGMIN8 subunit is the primary factor associating the AUGMIN complex to the parent microtubule and is hypothesized to set the angle and orientation for nucleation (Wu et al. 2008; Hsia et al. 2014; Lee et al. 2017; Song et al. 2018). Flowering plants have maintained a single copy of each gene encoding AUGMIN1‐7, while they have evolved a family of AUGMIN8 paralogs differentially expressed in mitotic and non‐mitotic cells (Albrecht et al. 2010; Pignocchi et al. 2009). Pioneering work showing that the AUGMIN8 paralog, EDE1 (QWRF5), is required for acentriolar plant spindle function (Lee et al. 2017), and further showing QWRF8 in cortical arrays of non‐mitotic plant cells, provides evidence that plants have conserved AUGMIN function for mitosis but have further extended AUGMIN function in non‐mitotic arrays.

Our experiments provide evidence for a modular QWRF family gene structure related to AUGMIN8 function with implications for AUGMIN complex regulation. Each of the five QWRF family members examined contains a monolithic 5′ exon with regions of shared homology, and regions with either no primary homology or weaker conservation between sub‐paralogous groups. The remainder of the 3′ gene is strongly conserved for coding sequence and intron structure (Figure 1). Protein sequence comparisons to animal AUGMIN8 proteins (i.e., HsHAUS8, XlHAUS8, and DmDgt4), indicate weak conservation across the N‐terminal regions encoded by the monolithic 5′ exons and strong conservation with the C‐terminal elements interacting with the AUGMIN complex. We note that the non‐mitotic plant QWRF proteins are larger than the animal AUGMIN8 orthologs due to an expansion of the N‐terminal region. The plant AUGMIN6 protein is reciprocally smaller than the animal orthologs, suggesting that microtubule association may be controlled more explicitly by AUGMIN8 in plants, without assistance from AUGMIN6 (Travis et al. 2023; Zhu et al. 2008; Zupa et al. 2022). Our microtubule localization data verifies that this first exon encodes a microtubule associating region for each paralog. Unexpectedly, the 3′ region of this monolithic exon appears sufficient to associate AUGMIN8 to AUGMIN6, indicating that binding to the parent AUGMIN complex is realized without the C‐terminal half of each paralog.

The *Arabidopsis* EDE1 (QWRF5) paralog is essential for plant mitosis and associates with mitotic spindle microtubules (Lee et al. 2017; Pignocchi et al. 2009). Attempts to rescue the loss of EDE1 (QWRF5) function, by QWRF8 expression under the EDE1 (QWRF5) promoter, failed (Lee et al. 2017). This result suggests that the function of the larger AUGMIN complex can be regulated through the interchange of different AUGMIN8 paralogs (Lee et al. 2017). Transient expression of EDE1 (QWRF5) in *N. benthamiana* leaf epidermal cells, driven into mitosis through cyclin‐D co‐expression, showed association with spindle microtubules (Xu et al. 2020). Contrary to prior data using an N‐terminal fluorescent fusion (Lee et al. 2017), we found that EDE1 (QWRF5) did not bind cortical microtubules in non‐mitotic cells. Reciprocally, we observed that full‐length QWRF4 and QWRF8 were mostly blocked from spindle microtubule association but readily colocalized with cortical microtubules in non‐mitotic cells. These experiments provide evidence that in the case of cell cycle phase, plant AUGMIN8 paralogs show regulation at the level of microtubule association, independent of association with the parent AUGMIN complex.

While full‐length QWRF4 and QWRF8 readily associated with cortical microtubules, full‐length QWRF1 and QWRF2 showed no consistent co‐localization. Expression of only the first exon from QWRF1 or QWRF2 in non‐mitotic cells produced weak, but consistent microtubule association, indicating that the N‐terminal microtubule association site we identified is functional in both proteins. We interpret these data to show that the full‐length QWRF1 and QWRF2 proteins require regulation, positive or negative, for cortical microtubule association in this heterologous system independent of the cell cycle regulation observed for EDE1 (QWRF5). These data, therefore, provide supporting evidence that specific plant AUGMIN8 proteins could be regulated to affect AUGMIN function beyond cases of mitotic and non‐mitotic arrays.

### 3.2 AUGMIN8 Association to Microtubules

Our in vivo analysis of the QWRF8 protein indicated two independent microtubule association domains. QWRF8 fragments including the first 125 AA produced robust microtubule co‐localization. We identified AUG8^26–88^ as a conserved domain across all QWRF family members and verified weak, but consistent microtubule association when expressed with the remainder of the N‐terminus. The “VxSRYxSPTPT” domain is strongly conserved across the other plant QWRF proteins. We note that the human, mouse, and *Xenopus laevis* HAUS8 genes share the “VxxSRY” motif between AA 34 and AA 42 suggesting some retention of the primary AUGMIN8 microtubule associating domain. Hence, we hypothesize that the association domain is not plant specific. While we cannot determine from the in vivo data if this protein sequence directly binds the microtubule lattice, we can predict that the sequence conservation across the QWRF family limits all family members to a common mechanism or site for microtubule association that must be differentially regulated between cell cycle phases.

We discovered a distinct second region in the N‐terminal half of QWRF8 sufficient for in vivo microtubule association. Using different fragment lengths, we narrowed the minimal sequence to AUG8^176–225^ in the in vivo system. We found little sequence conservation of this region with other *Arabidopsis* QWRF family members other than QWRF4. Comparisons across flowering plants (data not shown), however, indicate substantial conservation of this protein domain in at least one QWRF paralog per species, suggesting the second QWRF8 association domain is not unique to *Arabidopsis*.

The second QWRF8 microtubule association site appears weakly conserved in QWRF4. Efforts to evaluate in vivo binding suggest that QWRF4 can weakly associate with cortical microtubules in the absence of the primary association domain. Experiments to examine association at a second domain for QWRF1 and QWRF2 proteins were unsuccessful. We interpret these results to suggest that QWRF8 and QWRF4 have evolved a second microtubule association site and that QWRF1 and 2 either do not share this capacity or require some regulatory action not available in the transient system. We found that the first exon in the mitotic *EDE1 (QWRF5)* gene is substantially shorter than the other paralogs and has no recognizable sequence to test for a secondary microtubule association site. These observations provide evidence that the final footprint of different AUGMIN8 paralogs on the microtubule lattice likely differs. Our observation that QWRF8, and perhaps QWRF4, introduce a second microtubule association domain in this space suggests a functional difference in how the AUGMIN complex is anchored on cortical array microtubules.

### 3.3 Formation of Microtubule Associated QWRF‐Bodies

Prior animal studies presented evidence that more than one AUGMIN complex associates together on microtubules (Ustinova et al. 2023; Zhang et al. 2022). The rationale for the multi‐complex formation has not been reported. The full‐length QWRF8 and QWRF4 proteins exhibited discontinuous localization along all cortical microtubules on the cell face. Time‐lapse imaging of the full‐length proteins revealed subtle shifting of some sub‐resolution elements as well as accumulation on depolymerizing microtubule plus ends. We speculate that these observations are not likely to arise from single molecules and more probably denote shifting of larger assemblies. We found that co‐expression with full‐length AUGMIN6 did not change the discontinuous association pattern of QWRF8. Taken together, we propose that AUGMIN8 paralogs form larger molecular assemblies, independent of the larger AUGMIN complex, that lead to a discontinuous association along the microtubule lattice.

The discontinuous localization of QWRF8 along cortical microtubules shifts to continuous when the gene is truncated at a position just beyond the junction with the first 5′ exon. Expression of QWRF8 fragments without the first microtubule association domain (AUG8^150–644^) retained the discontinuous localization. From these observations, we hypothesize that the discontinuous localization arises from the local QWRF8 multimerization on the microtubule lattice and is not a property of how QWRF8 associates with the microtubules through the primary association domain (AS1). Consistent with this hypothesis, expressing QWRF8 fragments lacking the first microtubule association domain and extending for at least 100 AA (i.e., QWRF8^201–300^) produced fluorescent assemblies larger than the diffraction limit that retained microtubule association. The assembly size scaled roughly with increasing fragment length, ultimately creating larger “QWRF bodies” observed to move in the cell's cytoplasmic streams with episodic association with cortical microtubules. The changing size and microtubule association suggest that these assemblies are not simple aggregates and possess some properties of protein condensates. Fragments of QWRF2 that excluded the N‐terminal microtubule association site were also observed to form QWRF bodies, showing conservation of this property in other AUGMIN8 paralogs.

These observations suggest that there is an intrinsic capacity for the unstructured regions of QWRF8 to self‐assemble or, through multipartite binding, form multimers when associated to microtubules. Co‐expression of oppositely labeled full‐length QWRF4 with QWRF8 showed neither mutual exclusion nor obligate overlap in the microtubule localization. Both proteins retained a punctal or discontinuous localization along microtubules with no evidence for specific “hot‐spots” on the cell face or actively targeted regions of microtubule association. Together these data suggest that QWRF8 and QWRF4 show some one‐dimensional diffusion over short distances as multimeric assemblies and that microtubule associations are not restricted to specific regions of the cell or microtubule lattice. We speculate that when expressed at limiting concentrations for associating with the larger AUGMIN complex, the region of QWRF8 between AA300 and AA600 should be inaccessible for multimerization but could influence the spacing or multimerization of the AUGMIN complex prior to AUGMIN assembly.

### 3.4 Implications for Microtubule Patterning in Acentriolar Plant Cells

Acentriolar plant cells do not form conventional microtubule organizing centers (e.g., centrosomes), relying primarily on microtubule‐dependent microtubule nucleation (MDMN) at the cell cortex and undocumented mechanisms at the nuclear envelope to define where microtubules are nucleated (Erhardt et al. 2002; Murata et al. 2005; Nakamura et al. 2010; Seltzer et al. 2007). Cortical array microtubules remain laterally associated with the cell cortex over their lifetime and translocate via polymer treadmilling (Shaw et al. 2003). In turn, the treadmilling polymers interact to form co‐aligned arrays of both mixed and uniform microtubule polarity (Ehrhardt and Shaw 2006; Shaw et al. 2003). Observations of MDMN at the plant cell cortex show formation of branched and bundled microtubules created in both parallel and antiparallel arrangements (Chan et al. 2009; Shaw et al. 2003; Yagi et al. 2018). How plant cells, therefore, specify the position and orientation of new microtubules through nucleation, and how nucleation contributes to the formation of functional arrays at the cell cortex, remains central questions for plant cell biology.

Publicly available transcriptome data indicate broadly different expression patterns for the 9 QWRF family members. Coupled with the demonstrated requirement for EDE1 (QWRF5) in mitosis (Lee et al. 2017) and co‐localization of AUGMIN with nucleation in the plant cell cortex (Liu et al. 2014), these data suggest that different QWRF proteins are used with the AUGMIN complex to direct where, and possibly how, microtubules are nucleated through MDMN. Our data, using a heterologous system that preserves aspects of QWRF protein regulation, suggest a doubly modular system where different QWRF paralogs determine properties of the MDMN and the monolithic 5′ exon is the primary functional determinant of AUGMIN association to the parent microtubule. We propose that plant AUGMIN8 paralogs share one common microtubule association site closer to the N‐terminus and share a domain connecting the unstructured N‐terminal sequence to the conserved C‐terminal half of the protein that includes the “QWRF” motif. Between these conserved regions, we speculate that each QWRF paralog has evolved a different structure that alters how the AUGMIN complex associates with the parent microtubule, including the possibility of a second site of microtubule association.

## 4 Materials and Methods

### 4.1 Plant Materials

*Nicotiana benthamiana* seeds were surface‐sterilized using a 19:1 (v/v) solution of 87.5% EtOH:30% H_2_O_2_ on filter paper before sowing on 0.5× Murashige and Skoog medium (Murashige and Skoog Basal Salt Mixture, Sigma‐Aldrich) plates containing 1% agar (Sigma‐Aldrich). Plates were kept at 4°C for 2–5 days to synchronize germination, then transferred to a 24‐h light growth chamber. Plates were kept in the growth chamber for 11–12 days before planting seedlings.

### 4.2 Transient Expression

Plasmid constructs were transiently expressed in *Nicotiana benthamiana* leaf tissue by agrobacterial infiltration. *Agrobacterium tumefaciens* C58C1 carrying the plasmid constructs was grown in LB medium with the appropriate antibiotics at 30°C for 20–30 h. Cells were harvested by centrifugation, resuspended in infiltration buffer (10 mM MgCl_2_, 10 mM MES pH 5.6, 150 μM acetosyringone) and incubated at room temperature for at least 1 h. The bacterial resuspensions were mixed with other resuspensions carrying different constructs in a 1:1 ratio such that the final optical density (OD_600_) of each construct‐carrying bacteria was 0.2. The mixtures were infiltrated into 3–4‐week‐old *N. benthamiana* abaxial leaves. Leaf sections were imaged 2 days post infiltration.

### 4.3 Confocal Microscopy

*N. benthamiana* abaxial leaf epidermal cells were imaged using an Olympus OSR spinning disk confocal microscope with a 60×, 1.3 NA silicone oil immersion objective lens. The microscope used a Yokagawa W1 spinning‐disk confocal scanning unit with 50 μm pinholes. mNEON‐based probes were excited using the 488 nm laser line, and mScarlet‐based probes were excited using the 561 nm laser line. The emission was collected with a Hamamatsu Flash 4 V2 camera or Hamamatsu Fusion BT sCMOS camera.

## Conflicts of Interest

The authors declare no conflicts of interest.

## Supporting information


**Movie S1:** Association of QWRF‐bodies with microtubules. Construct expressing AUG8^201–644^‐mNEON was transiently expressed under the CaMV 35s promoter with 35s:mScarlet‐TUB1 in *N. benthi*. Time‐lapse images from double‐transformed leaf epidermal cell showing “QWRF‐bodies” (green) interacting with cortical microtubules. Time lapse images were collected at 3 s intervals for 1 min. Scale bar = 10 μm.

**Data S1:** Supplementary video caption.

## Data Availability

The data that support the findings of this study are available from the corresponding author upon reasonable request.
