# Supplementary material for: Arabidopsis AUGMIN8 Contains Two Independent Microtubule Association Domains
Source: Cytoskeleton (Hoboken). 2025 Nov 10;83(9):752–67. doi: 10.1002/cm.70068 (PMC13550708; doi:10.1002/cm.70068)
Supplement: Supplementary file 1 [file CM-83-752-s002.docx]

**Supplemental Movie** *Association of QWRF-Bodies with Microtubules.* Construct expressing AUG8^201-644^-mNEON was transiently expressed under the CaMV 35s promoter with 35s:mScarlet-TUB1 in *N. Benthi*. Time-lapse images from double-transformed leaf epidermal cell showing ‘QWRF-bodies’ (green) interacting with cortical microtubules. Time lapse images were collected at 3 second intervals for 1 minute. Scale bar = 10 um.
